# Comparison of Two Surgical Techniques for Periprosthetic Supracondylar Femoral Fractures: Minimally Invasive Locking Plate Versus Retrograde Femoral Nails

**DOI:** 10.14744/SEMB.2021.34270

**Published:** 2021-12-20

**Authors:** Samet Erinc, Necmi Cam, Muharrem Kanar, Haci Mustafa Ozdemir

**Affiliations:** Department of Orthopedics and Traumatology, University of Health Sciences Turkey, Sisli Hamidiye Etfal Training and Research Hospital, Istanbul, Turkey

**Keywords:** Locking plate, minimal invasive surgery, periprosthetic fracture, retrograde intramedullary nailing, supracondylar femoral fracture, total knee replacement

## Abstract

**Objectives:**

This study aimed to compare minimal invasive locking plate and retrograde intramedullary nailing in the treatment of supracondylar femur fracture following total knee arthroplasty (TKA) in respect of fracture healing, complications, and functional results.

**Methods:**

A retrospective analysis was made of 32 supracondylar femur fractures comprising 20 cases treated with minimal invasive locking plate fixation and 12 with retrograde femoral nailing. The two techniques were compared in respect of range of motion (ROM), functional scores, intraoperative blood loss, surgery time, and radiological examination findings.

**Results:**

The mean functional scores did not differ between the nailing and plate fixation groups. In the minimal invasive locking plate group, 2 (10%) patients had delayed union, so revision surgery was applied. The mean post-operative ROM was comparable between two groups (86.2° vs. 86°). Reduction quality in the sagittal plane and maintenance of the initial reduction were better in the minimal invasive locking plate group. Greater shortening of the lower extremity was seen in the retrograde femoral nailing group than in the minimal invasive locking plate group (20.3 vs. 9.3 mm). Perioperative blood loss was greater (2 units vs. 1.2 units) and mean operating time was longer in the minimal invasive locking plate group (126.5 min vs. 102.2 min).

**Conclusion:**

In patients with good bone stock, supracondylar femur fracture following TKA can be treated successfully with retrograde nailing or minimal invasive locking plate. Retrograde femoral nailing has the advantage of less blood loss and a shorter operating time. Reduction quality may be improved with the minimal invasive locking plate fixation technique. Both surgery techniques can be successfully used by orthopedic surgeons taking a case-by-case approach.

Supracondylar femur fracture after total knee arthroplasty (TKA) is a challenging situation in the elderly population. With longer life expectancy, the elderly population has grown, and so in the last few decades there has also been an increase in operations that are performed to promise a better quality of life. TKA is one of the most frequently performed procedures to respond to the high functional demands of the elderly population. The prevalence of TKA in the elderly doubled from 1991 to 2010 and is expected to further increase by 143% by 2050.^[1]^ The number of periprosthetic fractures resulting from traumatic injuries and osteoporosis is also likely to increase. The prevalence of supracondylar fracture after TKA ranges from 0.2% to 2%.^[2]^

There are several disadvantages specific to the treatment of supracondylar femur fracture following TKA. Poor bone stock, osteoporosis, and insufficient available bone for fixation due to the prosthesis and comorbidities of the patients are some of the challenging problems in periprosthetic fracture fixation. Although there is no consensus on the ideal treatment of periprosthetic supracondylar femur fracture, two types of fixation technique are currently used most: Locking plate and screw fixation and retrograde intramedullary nailing.^[3]^ Despite the many studies conducted to compare plate and retrograde intramedullary nailing, the treatment of periprosthetic supracondylar femur fracture is still controversial. Retrograde intramedullary nailing is a load-sharing device so provides greater stability and allows instrumentation through smaller skin incisions relative to the plate screw technique. However, achieving and maintaining fracture reduction with closed techniques may be more challenging than open reduction and plate screw fixation due to insufficient metaphyseal bone stock in osteoporotic patients. Other potential disadvantages of retrograde intramedullary nailing include prosthesis infection and knee stiffness. However, in plate fixation, the greater soft tissue and periosteal dissection can lead to a greater risk of non-union and periprosthetic fracture.^[4]^

The aim of this study was to compare the clinical and radiological results of retrograde intramedullary nailing and locking plate screw fixation of supracondylar femur fracture following total knee prosthesis.

## Methods

Approval for this retrospective study was granted by the Institutional Review Board (1431/11.02.2020). A total of 41 eligible patients were recruited from a scan of the electronic medical records including clinical progress and radiological examinations of patients admitted to our institution following a periprosthetic supracondylar femur fracture between February 2010 and January 2019. Inclusion criteria were retrograde intramedullary nailing or lateral based locking plate fixation of a supracondylar femur fracture following total knee prosthesis, patient age >65 years and minimum 12-month follow-up period. Patients were excluded if they had been applied with a knee prosthesis with long femoral stem, if they had a femoral fracture at the time of initial knee prosthesis implantation, a history of septic or aseptic prosthesis loosening, or a pathological or open fracture. After application of these criteria, 32 patients were included in the study. A total of nine patients were excluded from the study; four due to long femoral stem of TKA, three with a history of pathological fracture, and two due to mortality within 1 year postoperatively.

**Figure 1.**
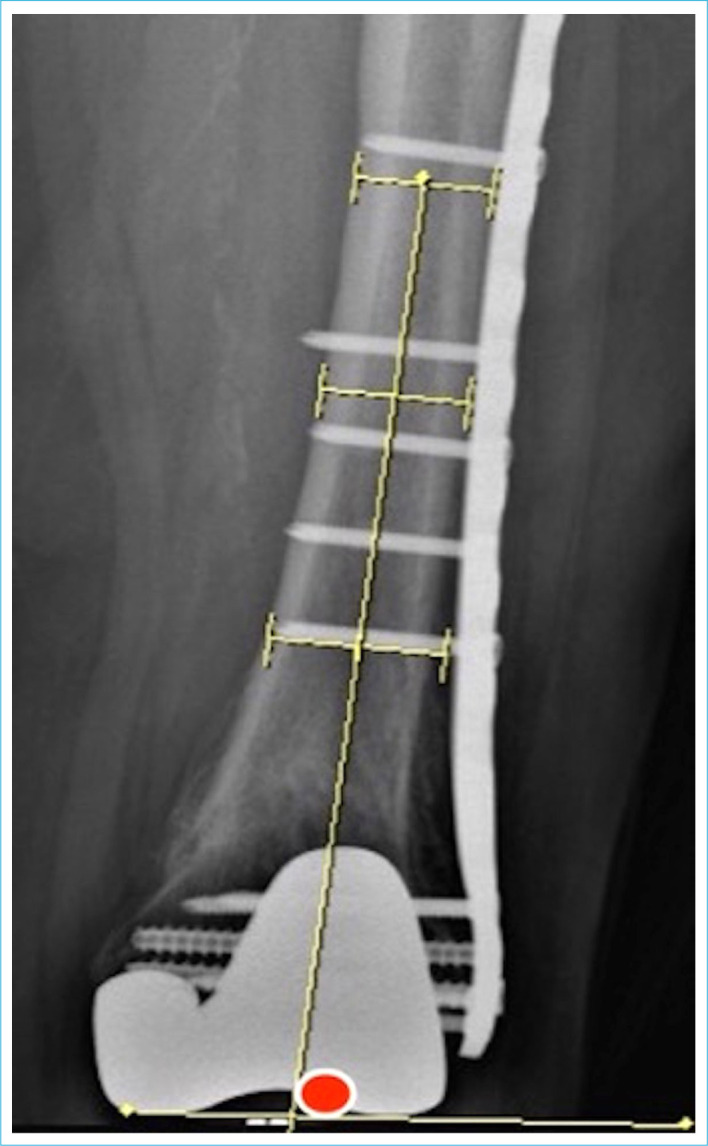
Measurement of the alpha angle.

**Figure 2.**
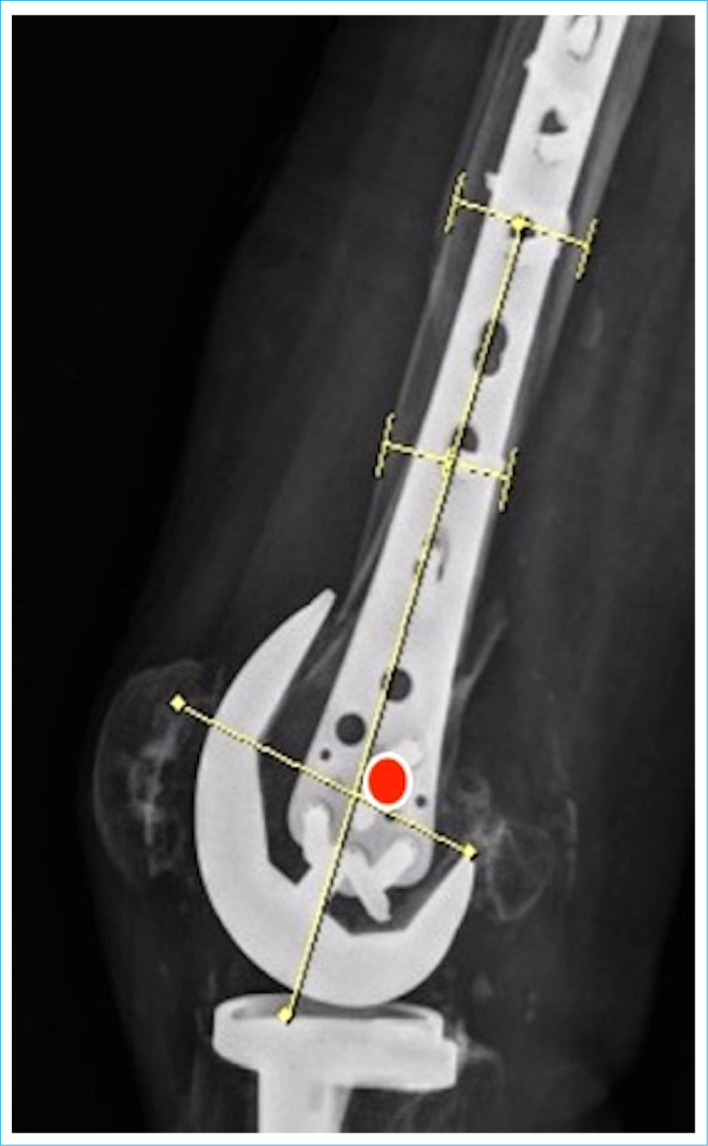
Measurement of the gamma angle.

**Figure 3.**
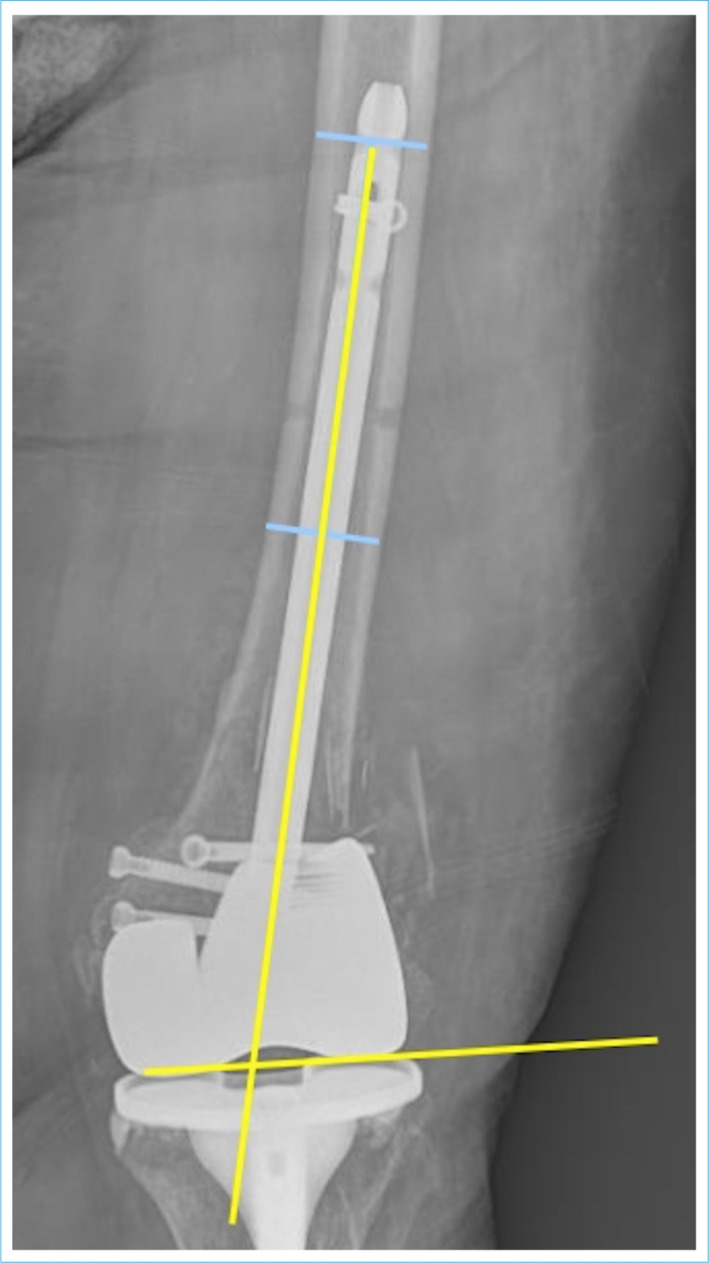
A – p X-ray of 72 year aged male patient with rIMN, 14.mos postoperatively. The alpha angle: 101°.

**Figure 4.**
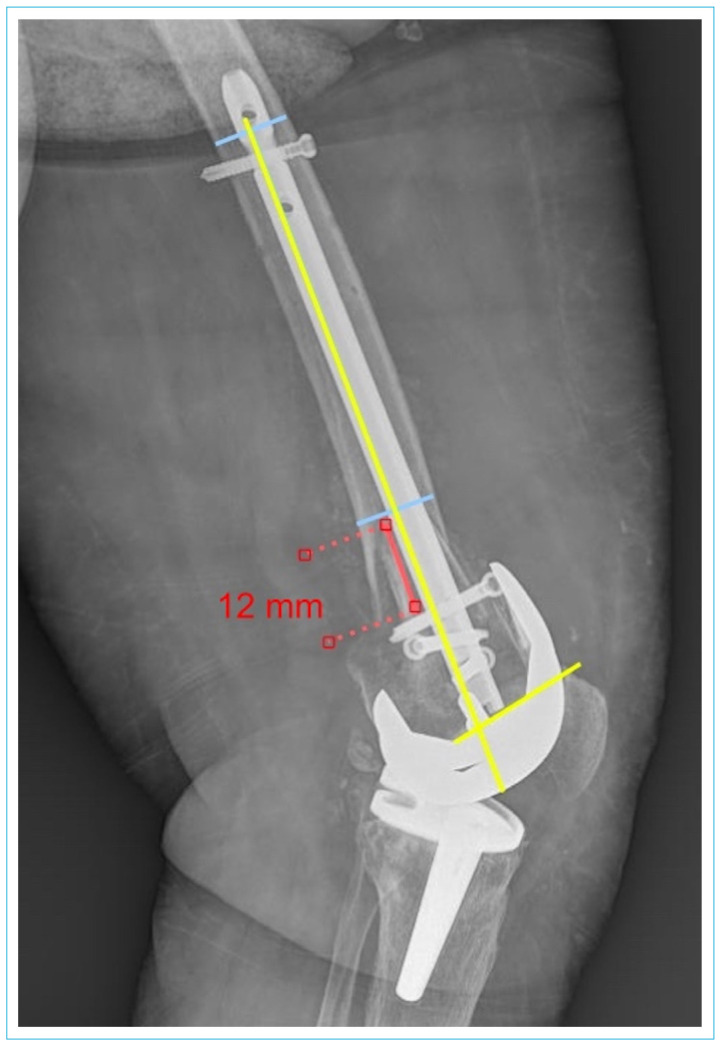
Lateral X-ray of the patient. The shortening was 12 mm, gamma angle: 80°.

The patients were divided into two groups according to the fixation technique; Group A, retrograde femoral nailing retrograde intramedullary nailing (IMNr), and Group B, lateral based locking plate minimally invasive plate osteosynthesis (MIPO). The following information was retrieved from the medical clinical records of the patients; range of motion (ROM) of the operated knee, time from TKA surgery to fracture fixation surgery, and time to union. The total operating room time and need for postoperative transfusion were recorded perioperatively, and postoperatively, complications such as infection, non-union, and need for revision surgery were noted. The shortening was determined with measurement of the telescoping of the fracture on the last X-ray obtained after fracture consolidation. Knee Society Score (KSS) and SF-12 scores were evaluated from clinical records of the last visit, with 80–100 KSS points representing excellent results, 70–79 good, 60–69 fair, and <60 poor.^[5]^ The Lane and Sandhu System was used for the assessment of radiological healing.^[6]^ Bone union was assessed with clinical and radiological examination. The fractures were classified according to the Rorabeck - Taylor classification and The Unified Classification System (UCS).^[7,8]^ Specifically two radiological parameters were used to evaluate the reduction; the alpha angle (lateral distal femoral angle) (Fig. 1), which is the lateral angle between the femoral shaft and the line through the distal part of the femoral component in the coronal plane (Normal: 87°±2°), and the gamma angle (Fig. 2), which is the angle between the femoral shaft and the line parallel to the distal part of the component in the sagittal plane (Normal: <10°, and ≥10° was defined as extension deformity).^[9]^ The quality of the initial reduction and its maintenance was assessed by the radiological parameters measured immediately postoperatively and again after the fracture consolidation (Figs. 3 and 4).

There were no signs of implant loosening at the time of injury according to the radiological examinations of the patients. After confirmation of the prosthesis stability, the fixation technique (IMNr or MIPO) was determined and applied by one senior surgeon and his surgeon team. The type of femoral component was the major issue in the determination of the fixation technique. In some prosthesis design, femoral box is not feasible for the entry point of the retrograde intramedullary nail. Ipsilateral total hip prosthesis was another drawback for intramedullary nailing. Retrograde femoral nailing was performed in the supine position on a radiolucent table under fluoroscopy. A support was placed under the knee to facilitate the fracture reduction and nail insertion. A medial parapatellar incision was used to find the appropriate entry point. After confirmation of the open – box design of the femoral component, the guide wire was entered in the intramedullary canal, and the canal was reamed up to 1 mm diameter more than the selected nail. The FN – 3 retrograde femoral nail (Tasarımmed, Istanbul, Turkey) was inserted retrograde in the intramedullary canal. Indirect reduction techniques such as joystick, percutaneous reduction clamps, and Kirschner wires were used for reduction of the fracture. Blocking screws were used in some cases to control varus – valgus and extension – flexion deformity. The minimally invasive plating was also performed in the supine position on a radiolucent table with a support placed under the knee. A lateral longitudinal incision was made at the level of the lateral epicondyle. A submuscular tunnel was prepared with a blunt elevator to insert the plate using the locking screw guide as a handle (MISS distal femur plate, TST Industries, Istanbul, Turkey). Under fluoroscopy guidance, the fracture was reduced with indirect reduction techniques as in the nailing technique. First the plate was secured with an unlocked screw to the proximal fragment and after confirmation of the reduction it was secured to the distal fragment. The number of locked screws was decided on an individual basis according to the fracture pattern. Control X-rays were taken immediately after the surgery, and monthly until fracture consolidation was observed.

In the early rehabilitation of patients with periprosthetic femur fracture there should be emphasis on ROM of the knee and weight-bearing as early as possible. Immediate mobilization is recommended in all patients. Full weight-bearing was allowed postoperatively with a walker. During this time, flexion and extension exercises were used to obtain full ROM of the knee.

### Statistical Analysis

Data obtained in the study were analyzed statistically using SPSS vn. 15.0 software. Descriptive statistics were stated as mean, standard deviation, median, minimum, and maximum values, frequency, and percentage. The Independent Samples t-test and the Mann–Whitney U-test were used for the comparison of quantitative data. The Chi-square test was applied in the comparisons of qualitative data. A value of P < 0.05 was accepted as statistically significant.

## Results

The results of the descriptive analyses are summarized in Table 1.

**Table 1.** Descriptive analyses

	**IMNr**	**MIPO**	**P**
Gender n (%)			
Male	3 (25.0)	14 (70.0)	>0.05
Female	9 (75.0)	6 (30.0)
AGE (years)			
Mean±SD	78.3±7.2	80.1±6.8	>0.05
(Min–Max)	(68–92)	(72–93)	
Etiology n (%)			
Primary	10 (83.3)	16 (80.0)	>0.05
Traumatic	2 (16.7)	4 (20.0)	
Type of TKA n (%)			
CR	5 (41.7)	9 (45.0)	>0.05
PS	7 (58.3)	11 (55.0)	
Mean interval time			
Mean±SD	30.5±7.9	28.8±8.4	>0.05
(Min–Max)	(16-42)	(10-42)	
Rorabeck n (%)			
1	5 (41.7)	5 (25.0)	>0.05
2	7 (58.3)	15 (75.0)	
UCS n (%)			
B1	12 (100)	20 (100)	>0.05

IMNr: Retrograde intramedullary nailing; MIPO: Minimally invasive plate osteosynthesis; TKA: Total knee arthroplasty; CR: Cruciate retaining, PS: Posterior stabilized; UCS: Unified classification system.

The IMNr group comprised 12 patients with a mean age of 78.3 years and the MIPO group comprised 20 patients with a mean age of 80.1 years. No statistically significant difference was determined between the groups in respect of age (p>0.05).

In the IMNr group, the mean interval from trauma to TKA was 30.5 months (range: 16–42 months). According to the UCS, all patients were classified as Type B1 and according to the Rorabeck classification, five patients (41.7%) were classified as Type 1 and seven patients (58.3%) as Type 2.

In the MIPO group, the mean interval from TKA to trauma was 28.8 months (range: 10–42 months). According to the Rorabeck classification, five patients (25%) were classified as Type 1 and 15 patients (75%) as Type 2.

The initial mean alpha angle in the IMNr group was 83.4 and 82.6 in the MIPO group, and the mean gamma angle was 7.8 in the IMNr group and 6.2 in the MIPO group, with no statistically significant difference between the two groups (p>0.05). At the time of the fracture healing, the mean alpha angle was 84.6 in the IMNr group and 83.2 in the MIPO group, with no statistically significant difference between the two groups (p>0.05). The mean gamma angle was 12.2 in the IMNr group and 6.8 in the MIPO group. The mean gamma angle was statistically significantly higher in the IMNr group than in the MIPO group in the 1^st^ year after surgery. In the IMNr group, there were six patients (50%) with a gamma angle ≥10, and in the MIPO group there were four patients (25%) with a gamma angle ≥10. Mean ROM of the knee joint was 86.2° in the IMNr group and 86.0° in the MIPO group with no significant difference determined between them (p>0.05) (Table 2).

**Table 2.** Radiological and clinical features of the patients

	**IMNr**		**MIPO**		**P**
	**Mean±SD**	**Min–Max (Median)**	**Mean±SD**	**Min–Max (Median)**	
Radiological parameters					
Postop. 1.day					
Alfa angle	83.4±2.4	77-97 (84)	82.6±2.2	75–92 (82)	>0.05
Gamma angle	7.8±2.5	2–14 (9)	6.2±4.3	0–21 (7)	>0.05
After fracture consolidation					
Alpha angle	84.6±3.6	78–96 (85)	83.2±1.6	76–93 (83)	>0.05
Gamma angle	12.2±4.6	5–20 (11)	6.8±6.2	0–24 (6)	<0.05
ROM	86.2±9.3	75–105 (84)	86.0±9.1	75–105 (85)	>0.05
KSS	67.7±12.7	46–86 (66)	69.5±14.7	34–92 (68)	>0.05
SF-12	63.8±12.6	30–82 (64)	67.2±15.5	30–92 (68)	>0.05
L-S SCORE	8.1±1.4	6–10 (8)	8.4±1.5	6–10 (8)	>0.05
Limb Shortening	20.3±7.6	9–30 (21)	9.3±6.6	0–26 (10.5)	<0.05
Union Time	4.2±0.9	3–6 (4)	4.3±0.9	3–6 (4)	>0.05
Mean Surgical Time (min.) n (Min–Max)	102.2±8.6	75–142 (101)	126.5±7.6	84 – 188(127)	<0.05
VAS Score	4.5±0.6	2–6 (4)	4.1±0.8	2–7 (4)	>0.05
Transfusion	1.2±0.6	0–2 (1)	2.0±0.7	1–3 (2)	<0.05

IMNr: Retrograde intramedullary nailing; MIPO: Minimally invasive plate osteosynthesis; ROM: Range of motion; KSS: Knee society score; L-S Score: Lane and Sandhu system score; VAS: Visual analog scale.

The mean clinical scores (KSS and SF-12) were higher in the MIPO group than in the IMNr group, but not to a statistically significant level. The mean KSS score was 67.7 points (range: 46–86) in the IMNr group, and 69.5 points (34–92) in the MIPO group. The mean SF-12 score was 63.8 points (range: 30–82) in the IMNr group, and 67.2 points (30–92) in the MIPO group. According to the KSS score, four patients had excellent results in the MIPO group and two patients in the IMNr group had a KSS score >80.

The mean Lane and Sandhu system score referring to radiological callus was 8.1 in the IMNr group and 8.4 in the MIPO group with no statistically significant difference between the groups (p>0.05). In the MIPO group, two patients (10%) required revision surgery in the postoperative 5^th^ month and 6th months for delayed union with allografting of the fracture site and locked plating re-fixation. No non-union was observed in the intramedullary nailing group No statistically significant difference was determined between the groups in respect of the Visual analog scale pain scores of the patients at 1 year after surgery (p>0.05). No statistically significant difference was determined between the groups in respect of the time to union at 4.3 months in the IMNr group and 4.3 months in the MIPO group (p>0.05). Mean lower extremity shortening was significantly greater in the IMNr group than in the MIPO group, at 20.3 mm and 9.3 mm, respectively (p<0.05). Shortening of >20 mm was observed in 3 (15%) patients in the MIPO group and 5 (41.6%) patients in the IMNr group. The mean amount of blood transfusion was statistically significantly more in the IMNr group than in the MIPO group (1.2 vs. 2 units) (p<0.05) (Table 2).

## Discussion

When the stability of the prosthesis is not endangered by the fracture, internal fixation of the fracture can be initially considered. According to the Rorabeck classification,^[7]^ type II periprosthetic fracture includes fractures with angulation >5° or displacement >5 mm with a stable knee prosthesis.^[10]^ Duncan and Haddad suggested a new classification system (UCS) for periprosthetic fractures. According to the UCS, type B1 represents a well-fixed implant.^[8]^ Although satisfactory outcomes have been reported for the surgical treatment of patients with supracondylar femur fracture following TKA, there is no consensus on the treatment strategy for these types of fractures.^[11]^

Minimal invasive locking plate systems have been shown to have biomechanical advantages compared to conventional plate constructs in patients with osteoporosis. Minimal invasive plate systems also have superiority with increased fatigue strength and increased ultimate failure load.^[12]^ Impressive results have been reported with the less invasive stabilization system (LISS) locking plate system for the treatment of periprosthetic supracondylar femur fracture. In 2001, Kregor et al. reported preliminary results of LISS plate fixation.^[13]^ A series of 11 periprosthetic supracondylar femur fractures were treated with LISS plate fixation and full union was obtained in all patients. Another study reported the results of Rorabeck Type II fractures treated with first-generation locking plates and it was stated that despite technical difficulty, the locking plate was an effective method for periprosthetic supracondylar femur fracture.^[14]^

Supracondylar femur fracture following TKA fixation with intramedullary nailing was first described with an anterograde technique by Hanks et al. in 1989.^[15]^ Jabczenski and Crawford published a preliminary report of four patients treated with IMNr.^[16]^ With advancements in intramedullary technology, the retrograde intramedullary nailing technique has become one of the most preferred treatment methods in patients with supracondylar femur fracture. Many retrospective studies have been published demonstrating excellent union results with good functional recovery.^[4,17]^ A biomechanical study simulated periprosthetic fracture in six pairs of human cadavers implanted with a PCL-retaining TKA. Based on bending and torsional moments, it was concluded that intramedullary nailing provides greater stability than plating.^[18]^ The main advantage of nailing is that it provides a more stable construct with minimal soft tissue stripping and preserves the local biology of the fracture site compared to plate fixation.^[19]^ However, intramedullary nails provide internal fixation of the fracture and osteoporotic cancellous bone may be biomechanically suboptimal for intramedullary fixation. High stress at the implant – bone interface may result in failure of fixation after intramedullary nailing of an osteoporotic metaphyseal fracture.^[20]^ An ipsilateral total hip arthroplasty is a contraindication for IMNr, otherwise, a stress riser is created at the implant junction. Distal bone stock and nail design should be considered for the placement of at least two or three distal interlocking screws.^[21]^ Prosthesis type is also important to identify whether the notch of the femoral component is suitable for entry into the intramedullary canal. All nails should be spanned to the level of the lesser trochanter and proximal interlocking should be placed to prevent “windshield wipering.”^[4]^

In the current study, evaluation was made of patients with supracondylar periprosthetic femur fracture treated with retrograde intramedullary nailing or minimal invasive plate fixation. The mean KSS score was 67.7 in the IMNr group and 69.5 in the MIPO group, both of which represent fair results, and there were no significant differences between the groups in respect of the KSS and SF-12 scores. The degree of soft-tissue injury or opening of the fracture site at the time of surgery has been associated with non-union.^[22]^ Developments in plate systems and the advances in minimally invasive techniques which enable minimal soft-tissue stripping and preservation of the periosteal blood supply have changed the surgical outcomes of the plate fixation technique. In the current study, there was no considerable difference between the groups in respect of bone healing time. The results of the current study were comparable with the results of several previous studies.^[3,19]^ Kilucoglu et al. published a study of 15 patients with periprosthetic supracondylar femoral fracture treated through retrograde nailing or a locked plate and reported no significant difference in time to healing and KSS between the two groups.^[23]^ Similar results were found in the study by Kyriakidis et al., in which it was stated that there was no significant difference in the meantime to union, functional scores and knee ROM between the patients treated with LISS plate and IMNr.^[24]^ Matlovich et al. compared two surgical techniques in the treatment of supracondylar femoral fracture and comparable results were found between the patients treated with intramedullary nailing or a locking plate in terms of time to union, and functional ROM. In contrast with the current study, there was reported to be no difference in fracture alignment between the two groups.^[21]^

The reduction quality was determined to be better in the MIPO group in the current study. Moreover, in the sagittal plane, there was better maintenance of the initial reduction with the use of a minimal invasive locking plate compared to the intramedullary nailing. The gamma angle ≥10° represents extension deformity of the TKA femoral component and the number of patients with extension deformity was higher in the IMNr group than in the MIPO group after the fracture consolidation. Although the mean loss of reduction in the coronal plane was negligible in both groups, the loss reduction in the sagittal plane was almost 50 and final mean gamma angle was 12.2° in the IMNr group. The mean shortening at the fracture site was 20.3 mm in the patients treated with retrograde intramedullary nailing, which was significantly greater than in the patients treated with locking plate (9.3 mm). Althause et al. published a study comparing four treatment methods for stabilization of supracondylar femur fracture following TKA and found no shortening or collapse in the fractures treated with less invasive plate while the patients treated with intramedullary nailing demonstrated 19 mm shortening and 10° extension collapse.^[25]^ Wick et al. compared LISS plates and retrograde nails in the treatment of supracondylar periprosthetic femoral fractures and found 18° valgus malunion in the nailing group. They also stated that LISS plate was a better implant than retrograde nailing for the treatment of fractures with a small distal fragment.^[26]^ In another study, Horneff et al. suggested that the LISS plate was a better device for the treatment of the periprosthetic femoral fractures due to longer fracture healing time in the intramedullary nailing group and better reduction maintenance in the LISS group.^[3]^ However, in the current study, no correlation was determined between extension deformity or shortening and decreasing clinical scores of the patients until the deformity did not exceed 15°. One subject in the IMNr group with 23° extension deformity had the worst KSS and SF-12 scores (46 and 30, respectively). ROM of the knee joint was also comparable in both groups with no statistically significant difference. When the pain scores of the patients were compared, there were no significant differences in the pain scores at 1-year postoperatively. The mean surgical time was longer and the amount of blood replacement was greater in the MIPO group, which was consistent with the findings of other studies.^[27-29]^

Recent meta-analyses have indicated no difference in the mean union time and functional scores between patients treated with locking plate or rIMN for a periprosthetic supracondylar femoral fracture.^[30]^ However, many complications have been reported such as delayed or non-union, infection, deep vein thrombosis (DVT), and pulmonary embolism.^[29]^ In the current study, there was no patient with infection, DVT, or pulmonary embolism. Delayed union was observed in 2 (10%) patients treated with LISS plate, and there were no patients with union problems in the rIMN group. Meneghini et al. reported 9% non-union or delayed union in patients with rIMN and 19% non-union or delayed union in patients with LISS plate.^[19]^ In a study by Hou et al., non-union was determined at 9% in the patients treated with LISS plate and one patient treated with rIMN had non-union due to infection.^[28]^ A total of 32 periprosthetic supracondylar femoral fractures treated with rIMN in three studies demonstrated excellent results with 100% union rate.^[17,31,32]^ However, meta-analyses have shown no significant difference in the union rate between the patients treated with locking plate or rIMN.^[33,34]^

This study had some limitations; primarily that it was retrospective in design with no randomization. Although the sample size was small, homogeneous distribution of the subjects strengthened the study. Another limiting factor is that the bone mineral density, which may be associated with loss of reduction and time to union, was not evaluated. Furthermore, assessment of full-length weight bearing X-ray radiograph of bilateral lower extremities is a more reliable method for the assessment of shortening, but in this study only the fractured extremity X-ray was used for the measurement. In addition, the assignment of patients was not mentioned due to the retrospective design of the study.

## Conclusion

The management of supracondylar femur fractures following TKA has unique and challenging drawbacks. The treatment modalities should be applied on a case-by-case basis in the elderly population due to comorbidities and variations in the medical history of the patients. The results of this study showed that supracondylar femur fractures following TKA with adequate bone stock could be treated successfully with either retrograde intramedullary nailing or minimal invasive locked plate. IMNr demonstrated superiority with a shorter operation time and lower perioperative blood loss, which may be beneficial for patients with a high risk of morbidity. Better reduction quality was achieved with the MIPO technique but the clinical scores and ROM of the knee joint indicated similar results in both groups.

### Disclosures

**Ethics Committee Approval:** Sisli Hamidiye Etfal Training and Research Hospital 02.03.2021/1827.

**Peer-review:** Externally peer-reviewed.

**Conflict of Interest:** None declared.

**Authorship Contributions:** Concept – S.E., H.M.Ö.; Design – S.E., N.C., M.K.; Supervision – H.M.Ö.; Materials – S.E., M.K.; Data collection &/or processing – N.C., M.K.; Analysis and/or interpretation – S.E., N.C., M.K.; Literature search – S.E., N.C.; Writing – S.E.; Critical review – H.M.Ö.
